# Knowledge of Lassa fever and use of infection prevention and control facilities among health care workers during Lassa fever outbreak in Ondo State, Nigeria

**DOI:** 10.11604/pamj.2018.30.56.13125

**Published:** 2018-05-24

**Authors:** Ibidolapo Taiwo Ijarotimi, Olayinka Stephen Ilesanmi, Adeola Aderinwale, Oluwadamilola Abiodun-Adewusi, Ime-Maria Okon

**Affiliations:** 1 Nigeria Field Epidemiology Training Program, 50 Haile Selassie, Asokoro, Abuja, Nigeria

**Keywords:** Lassa fever, infection prevention and control, health centres, health care workers

## Abstract

**Introduction:**

Hospital-acquired infections of Lassa fever (LF) have been described in many West African countries. We assessed the availability of Infection Prevention and Control (IPC) measures and their use in the health centres (HCs) at the affected Local Government Areas (LGAs) during an ongoing LF outbreak in Ondo State, Nigeria.

**Methods:**

We included all primary and secondary HCs and their healthcare workers (HCWs) in the affected Ose and Owo LGAs. We collected data from respondents using self-administered questionnaires and used a checklist to assess the IPC measures at the HCs. We generated frequencies and proportions and tested associations using odds ratios at 95% CI.

**Results:**

One hundred and ninety HCWs from 59 HCs were surveyed of which 34 (57.6%) were located in Owo LGA. All HCs had soap for handwashing, 57(96.6%) had wash-hand basins but only 52(88.1%) had water. While 57(96.6%) had gloves and 53(89.8%) had sharps boxes, only 16(27.1%) had an isolation room. Only 44(23.2%) respondents had been trained in IPC. The majority, 144 (91.6%) always had gloves available for their use, 79(41.6%) always had facemask/shield and 71(37.4%) always had full personal protective equipment. At the last patient contact, only 151 (79.8%) washed their hands before the contact, 188(98.9%) washed their hands after and 183 (96.2%) wore gloves. While there was no association between availability of gloves and its use (OR: 0.21, 95%CI 0.04-1.17), there was significant association between having had training in basic universal precautions and having used gloves (OR: 3.64, 95%CI 1.21-19.40) and having washed hands after last patient contact (OR: 2.31, 95%CI 1.67-12.30).

**Conclusion:**

Among these HCs that serve as point of first contact with possible cases of LF in these endemic LGAs, none met the minimum standard for IPC. We recommend that IPC committee for each LGA and the whole state should be set up and IPC trainings made mandatory.

## Introduction

Lassa fever is a zoonotic disease caused by an arenavirus, the lassa virus, so named after the town in Nigeria where it was first isolated [1]. Humans contract the virus primarily through contact with the contaminated excreta of Mastomys natalensis rodents (commonly known as the Multimammate rat), which is the natural reservoir for the virus. The infected rodents are reservoirs capable of excreting the virus through urine, saliva, excreta and other body fluids to man [2]. Secondary transmission of the virus between humans occurs through direct contact with infected blood or bodily secretions. This occurs mainly between individuals caring for sick patients although anyone who comes into close contact with a person carrying the virus is at risk of infection. Incubation period of Lassa fever is 1-3 weeks. It presents with symptoms and signs indistinguishable from those of febrile illnesses such as malaria and other viral haemorrhagic fevers such as Ebola. In approximately 80%, symptoms are mild and are often undiagnosed. Death may occur within two weeks after symptom onset due to multi-organ failure [3]. While approximately 15%-20% of patients hospitalized for Lassa fever die from the illness, only 1% of all lassa virus infections result in death [4].

Nosocomial transmission of Lassa fever in healthcare facilities represent a significant burden on the healthcare system [5]. Infection prevention and control (IPC) in healthcare settings has been documented as an important factor in controlling potential outbreaks of Lassa fever [6]. In support of this, studies have shown that in hospitals with improved IPC practices, transmission of Lassa virus was minimal [7,8]. Lassa fever is endemic in parts of West Africa including Sierra Leone, Liberia and Nigeria; affecting about 100,000 to 300,000 people every year in this regions [9, 10]. There have been several Lassa fever outbreaks in various parts of Nigeria since it was first reported in 1969. The worst outbreak was recorded in 2012 when 623 cases including 70 deaths were reported from 19 out of the 36 states. Three doctors and four nurses were reported to be among the fatalities [11]. Between August 2015 and 17 May 2016, WHO was notified of 273 cases of Lassa fever reported from 23 states in Nigeria; these included 149 deaths. Of these, ten cases were health care workers (HCWs) and four were hospital acquired infections [11]. Many Local Government Areas in Ondo State have yearly outbreaks of Lassa fever which often affect health workers who are particularly at risk in resource poor settings like Nigeria due to lack of availability and use of IPC materials. Conscientious use of IPC during patient care irrespective of diagnosis and the use of transmission specific IPC materials when caring for patients with Lassa fever reduces the risk of hospital based transmission. The aim of this study was to assess the knowledge of Lassa fever among health care workers, availability of infection prevention and control measures and their use during Lassa fever outbreak in Ondo State.

## Methods

**Study design**: We conducted a descriptive cross sectional study among HCWs in Owo and Ose LGAs.

**Study area**: We purposively selected Owo and Ose LGAs because they were experiencing an outbreak at the time of this study. The two LGAs are the boundaries of the State with neighbouring Edo State which is known to be endemic for Lassa fever. Ondo state, with Akure as the capital, consists of 18 LGAs. The population was 3,441,024 as at 2006 census. Majority of the state´s citizens live in urban centers [12].

**Study population**: We carried out the study among HCWs who had been working in either secondary or primary health centers in the selected LGAs for at least six months prior to the commencement of our study.

**Sampling**: We did a total sampling of all primary and secondary health centers (both government and private owned) and their HCWs in the selected LGAs.

**Data collection**: We administered semi-structured interviewer administered questionnaire to the HCWs to assess their knowledge on Lassa and their practice of IPC. We used a checklist to assess the availability of infection prevention and control measures in the Health centers.

**Data management**: Data was entered and analyzed using Statistical Package for Social Sciences version (SPSS) 23 and Microsoft Excel 2016 software. Results were presented in frequencies, means and proportions. Each rightly answered question on knowledge was scored one and wrong answers scored zero. The scores were summed, seventy percent (70%) of the total score was used as the cut off between good and poor knowledge. Maximum achievable score for knowledge on epidemiological features of Lassa fever among all respondents was 11 and minimum zero. Maximum achievable score on knowledge of the clinical features of Lassa fever was 15 and minimum zero. Maximum achievable score for knowledge of precautions to be taken while caring for Lassa patients was eight and minimum zero. Associations between knowledge and availability of measures and practice were tested using chi square or Fishers test, where appropriate, with the level of significance set at 5%.

**Ethical considerations**: We obtained ethical approval from the Ondo State Hospital Management Board Ethical Review Board. We obtained informed consent from the respondents. We made them to understand that participation was voluntary and there was no consequence for non-participation. All information obtained were kept confidential.

## Results

Fifty-nine health facilities and one hundred and ninety healthcare workers were surveyed. Of the 59 health facilities surveyed, 9 (15.3%) were secondary health facilities the rest were primary. Of the 190 healthcare workers, 144 (75.8%) worked at primary health facilities while 155 (81.6%) worked at Government-owned facilities (Table 1).

**Table 1:** Sociodemographic characteristics of respondents, Ose and Owo LGAs, Ondo State, 2016

Sociodemographic characteristics	Ose (n=74)	%	Owo (n=116)	%	Total (N= 190)	%
**Sex**						
Male	16	21.6	19	16.4	35	18.4
Female	58	78.4	97	83.6	155	81.6
**Mean age (in years)**	28.8(±9.0)		33.7(±10.7)		31.8 (10.3)	
**Religion**						
Christianity	65	87.8	109	88.8	174	91.6
Islam	9	12.2	7	6.0	16	8.4
**Ethnicity**						
Yoruba	68	91.9	103	88.8	171	90.0
Others^#^	6	8.1	13	11.2	19	10.0
**Profession**						
Doctor	0	0	1	0.9	1	0.5
Registered nurse	12	16.2	21	18.1	33	17.4
Community Health worker	41	55.4	55	47.4	96	50.5
Auxiliary nurses	11	14.9	13	11.2	24	12.6
Health assistants	5	6.7	9	7.7	14	7.4
Others*	5	6.8	17	14.7	22	11.6
**Facility ownership**						
Government owned	59	79.7	96	82.8	155	81.6
Private owned	15	20.3	20	17.2	35	18.4
**Facility tier**						
Secondary	20	27.0	26	22.4	46	24.2
Primary	54	73.0	90	77.6	144	75.8

# Ibo and Edo

* Pharmacists and Laboratory scientists and technicians

### Healthcare workers

Of 190 respondents, 44 (23.2%) worked at Ose LGA and 35 (18.4%) were males. Mean age was 31.8±10.3 years. They were mostly Christians (174, 91.6%) and mainly Yoruba (171, 90%). Half (96, 50.5%) of them were Community Health Workers, 33 (17.4%) were registered nurses, 24 (12.6%) were auxiliary nurses or auxiliary trainee nurses and 1 (0.5%) was a doctor (Table 1). Among the respondents, only 44 (23.2%) have had a training in infection prevention and control (IPC).

### Overall knowledge

The overall knowledge score achieved by all respondents was 24.3±4.0. Less than half, 79 (41.6%) of respondents had good overall knowledge of Lassa fever epidemiology, clinical features and precautions to be taken in caring for affected patients. Registered nurses had better knowledge compared to auxiliary nurses/trainees (OR: 10, 95% CI: 5.06-15.0), Health Assistants (OR: 5, 95%CI: 1.27-19.6), Community Health Workers (OR: 2.5, 95% CI: 1.07-5.6), and other cadres of health workers (OR: 5.6, 95%CI: 2.74-12.5). Those that have had training in IPC had better knowledge than those who had not (OR: 3.9, 95% CI: 1.8-8.2).

### Knowledge of epidemiology of Lassa fever

**Figure 1:**
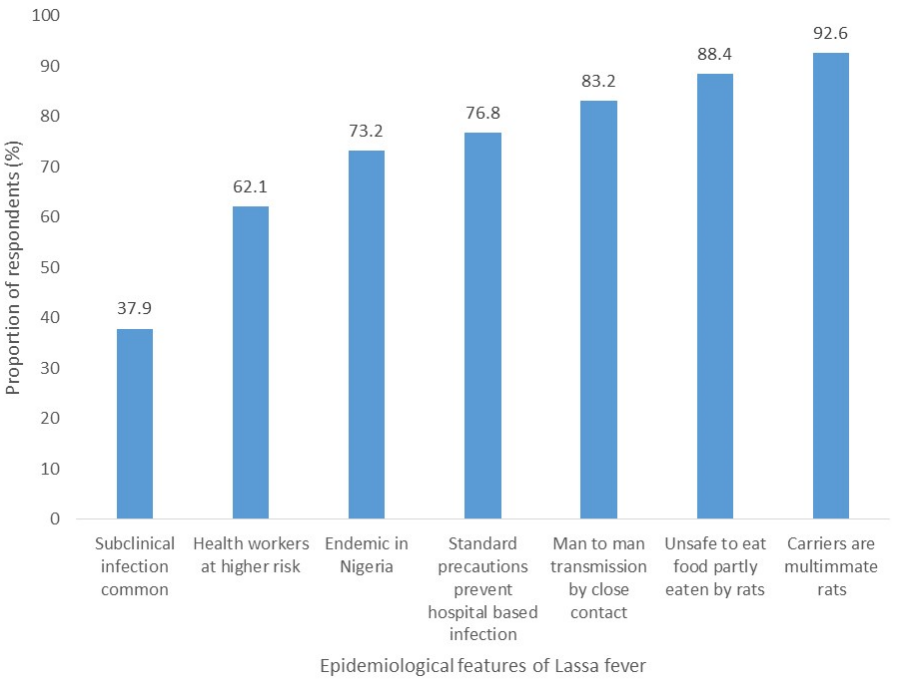
Proportion of HCWs that correctly identified each of the epidemiological features of LF, Ose and Owo LGA, Ondo State, 2016

The mean score on knowledge of epidemiology of Lassa fever among all respondents was 6.8 (±1.5). There was a statistically significant difference between the mean score among respondents from government owned facilities (mean score = 6.9 ±1.3) and private (mean score = 6.2 ±2.2) (p = 0.015). Only 26.8% had good knowledge and there was no statistically significant relationship with profession (p=0.325), facility ownership (p = 0.474) and LGA (p=0.413) (Figure 1).

### Knowledge of clinical features

**Figure 2:**
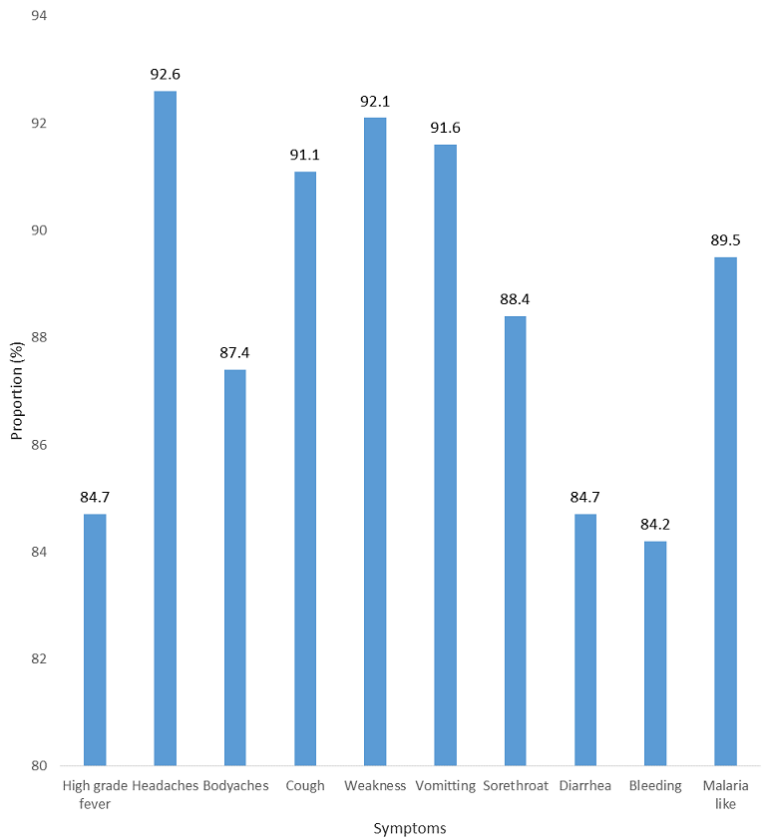
Proportion of HCWs that correctly identified each symptom of LF, Ose and Owo LGAs, Ondo state, 2016

The mean score achieved by all respondents was 11.4±2.4. There was a statistically significant difference between the mean score among respondents in government facilities (11.6±2.1) and private (10.4±3.2) (p = 0.009). Only 100 (52.6%) of all respondents had good knowledge of clinical features (Figure 2). There was a statistically significant relationship with LGA (p = 0.002). Majority (125, 65.8%) knew it was not 100 % fatal while about a third (58, 30.5%) knew deafness was a sequela.

### Knowledge of precautionary measures (Figure 3)

Mean knowledge score achieved by all respondents was 6.2 ±1.6. There was a significant association between mean knowledge scores of respondents from government facilities (mean score=6.4±1.4) and those from private (mean score= 5.5±1.6), (p=0.03).

**Figure 3:**
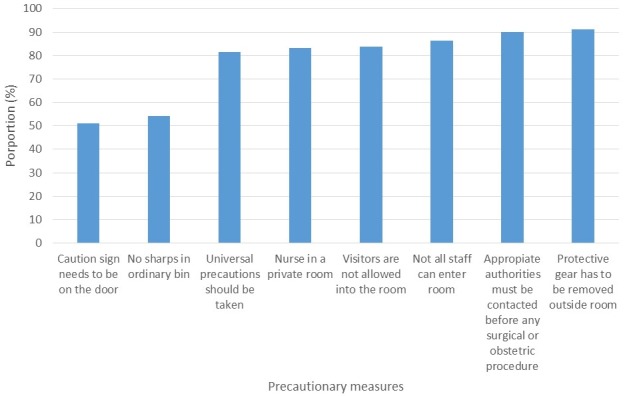
Proportion of HCWs who recognized appropriate precautionary measures to be taken while caring for a LF patient, Ose and Owo LGAs, 2016

### Availability of Personal Protective Equipment (PPEs) at work place

**Figure 4:**
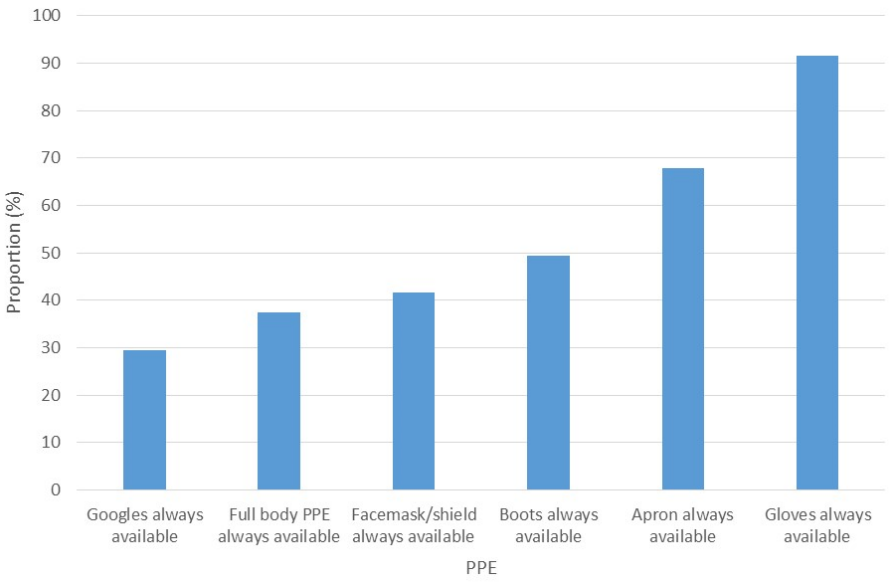
Availability of PPEs to HCWs, Ose and Owo LGAs Ondo state, 2016

In all, 174 (91.6%), always had gloves available for their use, 79 (41.6%) always had facemask/shield always available, and less than a third, 56 (29.5%), always had googles available. About two-thirds, 129 (67.9%), always had apron available and almost half 94 (49.5%) always had boots available, while 71 (37.4%) always had full body PPE available (Figure 4).

### Practice of basic universal precautions

**Figure 5:**
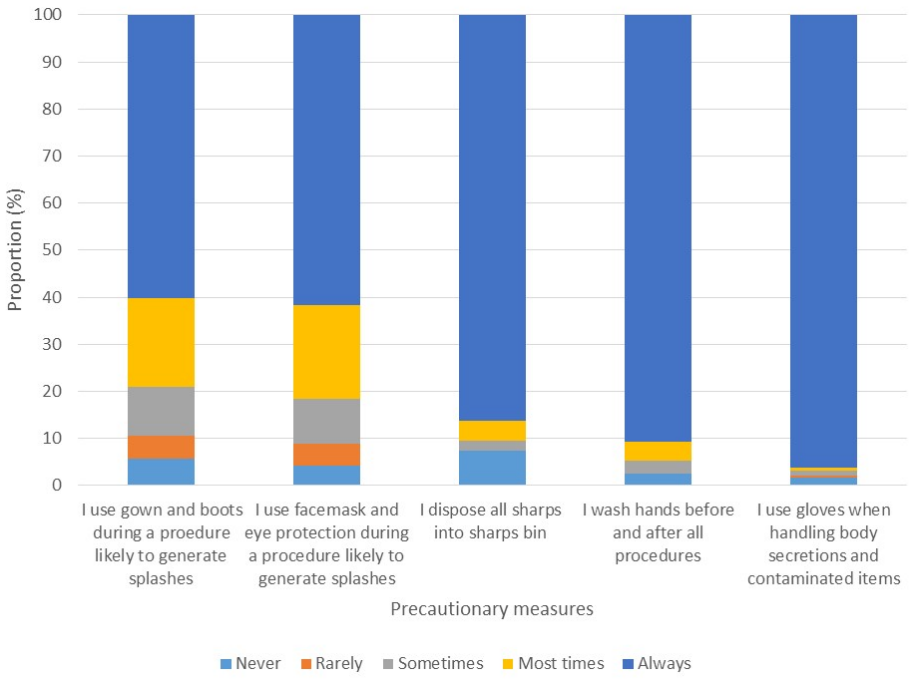
Use of precautionary measures during patient care by HCW in Owo and Ose LGAs, Ondo state, 2016

About 183 (96.3%) reported that they routinely used gloves when handling body secretions and contaminated items while, 171 (90.5%) reported that they routinely washed hands before and after all procedures. Only 86.3% routinely disposed all sharps into sharps bin. Sixty percent of respondents routinely used gown and boots during a procedure likely to generate splashes while 116 (61.1%) routinely used facemask and eye protection during such procedures as well. At the last patient contact, only 152 (79.8%) washed their hands before the contact, 188 (98.9 %) washed their hands after and 183 (96.2%) wore gloves (Figure 5). While there was no association between availability of gloves and its use (OR: 0.21, 95%CI: 0.04-1.17), health workers who have had a training in IPC were more likely to have used gloves at the last patient contact (OR: 3.64, 95%CI 1.21-19.40) and they were also more likely to have washed their hands at the last patient contact (OR: 2.31, 95%CI 1.67-12.30).

### Health facilities

All 59 had soap for hand-washing, but 7 (11.9%) didn’t have water out of which 6 (85.7%) were government-owned facilities and all were primary facilities. Wash-hand basin was available in 57 (96.6%) and 52 (88.1%) had water available. Two (3.4%) of the facilities didn’t have gloves. Only 20 (33.9%) had chlorine solution, 52 (87.2%) of these were government-owned facilities and primary facilities respectively. No health facility had all Infection Prevention and Control (IPC) requirements (Table 2).

**Table 2:** Facility ownership of IPC measures by facility tier, Ondo State, 2016

Characteristic	Secondary N=9	%	Primary N=50	%	Fishers P Value
Had isolation ward or room	4	44.4	12	24.0	0.236
Had changing room	7	77.8	29	58.0	0.460
Had restricted access room	4	44.4	10	20.0	0.195
Had soap for hand-washing	9	100	43	86.0	N/A
Had water	9	100	48	96.0	0.581
Had wash hand basin present	9	100	44	88.0	1.000
Had sharps box	9	100	16	32.0	0.577
Had chlorine solution	4	44.4	48	96.0	1.477
Had gloves	9	100	48	96.0	1.000
Had face shield or goggles	9	100	33	66.0	0.471
Had apron	9	100	12	24.0	0.480
Had full body PPE	3	33.3	25	50.0	0.680
Had boots	6	66.7	38	76.0	0.477
Had red color-coded waste bin	4	44.4	34	68.0	0.103
Had black color-coded waste bin	5	55.6	28	56.0	0.471
Had yellow color-coded waste bin	2	22.2	23	67.6	0.800

## Discussion

General knowledge of Lassa fever was low among health workers in the Lassa fever endemic LGAs under study. Particularly, the knowledge of the clinical features of the disease was low despite the fact that early diagnosis and treatment with ribavirin contributes significantly to survival from Lassa fever [9]. This could mean that even if patients with Lassa fever present early at health facilities, they may not be diagnosed and receive appropriate treatment on time. This finding of low knowledge of Lassa fever is in contrast to that of Omotowo et al and Adebayo et al, both of which were conducted among healthcare workers in tertiary facilities in Nigeria. The difference in the results however, maybe due to the fact that doctors and nurses constituted the majority of their respondents and the studies were conducted at tertiary health facilities that were specialised in caring for Lassa fever cases [13,14]. However, the study by Aigbiremolen which was conducted among staff of primary health facilities only and used 75% as a cut off, found that almost 80% of the respondents had good knowledge [15]. The same study found that Community Health Extension Workers (CHEWs) had poorer knowledge of Lassa fever than trained nurses as this study did [15]. Therefore, the low knowledge found in this study could also be due to the high proportion of CHEWs and trainee auxiliary nurses [16]. However, other studies have also documented poor knowledge among other cadres of health workers (16).

A similarly low knowledge was found by the study of Ekuma and Akpan which was conducted in a tertiary facility among medical students, interns and resident doctors in Uyo [17]. Therefore, it is also possible that other factors apart from profession and level of healthcare practice influence the acquisition of knowledge on Lassa fever. In addition, while a study by Olowookere et al found an association between ownership of health facility and knowledge of Lassa fever [18], we did not find a significant relationship. The study by Aigbiremolen did find that workers at private HCs had better knowledge than the workers at government owned PHCs though this relationship was also not significant [15]. Knowledge of precautionary measures was on the average and comparable with that of the findings of Izegbu et al in Lagos although there’s was conducted among laboratory staff only [19]. While the use of PPE was also comparable with that of the findings of Adebayo et al [14]. According to Adewuyi et al, the most dangerous exposure is parenteral and must be avoided through staff training [20]. Unfortunately, the result of the study shows that less than a quarter of the healthcare workers in these LGAs that are endemic for Lassa fever have had any training on IPC. Training of health workers on priority diseases is of importance [21]. The recommendation is for such patients to be treated in isolation rooms with adequate PPEs available at all times [22], our study found that none of the secondary health facilities had neither an isolation room nor have PPEs routinely available.

Concerning the routine practice of IPC measures, different studies have found varying levels of IPC practice in different parts of the country similar to what was found by this study [15, 23-27]. The consistent finding in all the study is that the practice of IPC was poor irrespective of the level of health facility. While it was not the focus of this study, it was however found that there was a lack of qualified health personnel at the primary and secondary health facilities that were studied. Community Health Workers (CHEWs) constituted majority of the workforce in the facilities. There was also a high proportion of auxiliary nurses/trainees. However in Nigeria, CHEWs are expected to predominantly be at the primary health care level [28,29], this is not supposed to be so at secondary health care level. This pattern seen here is probably because there is lack of incentives for qualified personnel to work at primary and secondary healthcare facilities especially those located in semi-rural or rural places. This further proves that we still have a mal-distribution of healthcare workers in the country [30]. A major limitation of this study was a probable high proportion of acceptable IPC practices among the respondents than usual as these were self-reported and thus prone to bias.

## Conclusion

This study found that the knowledge of HCWs about Lassa fever, its management and the IPC practices at health facilities in the LGAs studied were still inadequate therefore putting the HCWs at risk of hospital based transmission of not only Lassa fever but other infections. Ondo State Government should ensure HCWs receive refresher training on IPC regularly, and also set up IPC committees in each LGA that will ensure all health facilities in the LGAs meet the necessary IPC requirements.

### What is known about this topic

- Hospital-acquired infections of Lassa fever has been described in many West African countries;
- Nosocomial transmission and outbreaks in healthcare facilities in endemic areas represent a significant burden on the healthcare system;
- Infection prevention and control (IPC) in healthcare settings is an important factor in controlling potential outbreaks of Lassa fever.

### What this study adds

- The knowledge of Lassa fever among health care workers;
- Availability and use of infection prevention and control measures during Lassa fever outbreak in Ondo State, Nigeria.

## Competing interests

The authors declare no competing interests.
